# Impacts of Climate Warming on the Body Composition of Patients Undergoing Maintenance Hemodialysis

**DOI:** 10.7150/ijms.101232

**Published:** 2024-10-21

**Authors:** Wen-Fang Chiang, Po-Jen Hsiao, Kun-Lin Wu, Ruei-Lin Wang, Chi-Ming Chu, Jenq-Shyong Chan

**Affiliations:** 1 Division of Nephrology, Department of Internal Medicine, Armed Forces Taoyuan General Hospital, Taoyuan, Taiwan.; 2 Division of Nephrology, Department of Internal Medicine, Tri-Service General Hospital, National Defense Medical Center, Taipei, Taiwan.; 3 Department of Life Sciences, National Central University, Taoyuan, Taiwan.; 4 Division of Biostatistics and Medical Informatics, Department of Epidemiology, School of Public Health, National Defense Medical Center, Taipei, Taiwan.; 5 Graduate Institute of Medical Sciences, National Defense Medical Center, Taipei, Taiwan.

**Keywords:** bioimpedance analysis, body composition, climate change, dialysis, fluid overload

## Abstract

**Background:** Climate change, with increasing temperatures, poses a health threat to patients on maintenance hemodialysis (MHD). Seasonal variations in body composition have been documented in this population. We hypothesized that climate warming could further exacerbate these effects. In this study we investigated the impact of climate warming on the body composition of MHD patients residing in subtropical Taiwan.

**Methods:** This longitudinal observational study enrolled MHD patients in subtropical northern Taiwan. We assessed monthly blood pressure (BP), laboratory data, and body composition via bioimpedance spectroscopy over a three-year period. Generalized estimating equation (GEE) was employed to analyze the seasonal and annual variations in these parameters. Additionally, we explored associations between climatic variables and body composition parameters.

**Results:** Forty patients completed the study. BP, laboratory values, and body composition exhibited significant seasonal variations. Compared with those in winter, participants had greater relative overhydration (OH) in spring, summer, and fall. Warmer months were associated with a higher lean tissue index (LTI) and a lower fat tissue index (FTI). Notably, summers across the study years showed a further increase in relative OH and FTI, accompanied by a decrease in LTI. While BP and most laboratory parameters remained stable throughout the study period, sodium and potassium levels displayed annual variations. GEE analysis revealed positive associations between rising ambient temperature and increased fluid overload, fat mass, and decreased muscle mass.

**Conclusions:** Our findings demonstrate that climate warming is associated with variations in the body composition of MHD patients residing in a subtropical climate. These changes can have implications in MHD patients due to their heightened vulnerability to environmental changes. Further research is needed across diverse geographic regions to develop optimal care strategies in a warming world.

## Introduction

Climate change, characterized by a global average surface temperature increase of approximately 1.1°C since 1900, with an accelerated rate in the past 50 years, poses significant threats to public health 1. This impact is felt directly through increased heatwaves, leading to heat-related illnesses and deaths, particularly among vulnerable populations such as elderly individuals, children, and those with preexisting health conditions 2. Extreme weather events such as hurricanes, floods, and wildfires cause injuries, fatalities, and displacements and disrupt healthcare services, further worsening health outcomes. Indirectly, warmer temperatures expand the habitats of disease-carrying vectors, increasing the spread of diseases such as malaria and dengue 3. Changes in precipitation and flooding heighten the risk of waterborne diseases and food insecurity. Additionally, poor air quality exacerbated by higher temperatures worsens respiratory conditions such as asthma 4. Mental health is also affected, with increasing anxiety, depression, and trauma linked to the stress of extreme weather events and displacement 4.

End-stage renal disease (ESRD) patients are particularly susceptible to these climate change effects because of their compromised health status. Higher temperatures increase the risk of heat-related illnesses, cardiovascular events, and complications arising from fluid and electrolyte imbalances, which are especially dangerous for individuals with restricted fluid intake and preexisting cardiovascular disease. Studies have shown that exposure to extreme heat increases the risk of hospital admission or mortality in ESRD patients 5. These patients are also more vulnerable to infections, poor air quality, and mental health stresses. Overall, climate change exacerbates the complexity and cost of managing ESRD patients, highlighting the need for healthcare providers and policymakers to develop strategies to protect this vulnerable population.

Body composition plays a crucial role in managing ESRD patients. It provides insights into their nutritional status and fluid balance, both of which significantly impact cardiovascular health and treatment outcomes 6, 7. Various conditions, including protein-energy wasting, chronic inflammation, and acute infectious diseases, can alter body composition, particularly by causing loss of lean tissue mass and fluid overload in these patients. Previous studies have shown seasonal variations in body composition in patients undergoing maintenance hemodialysis (MHD). Specifically, fluid overload and muscle mass tend to increase during warmer months, while fat mass tends to increase during colder months 8. These findings suggest a potential correlation between body composition and climatic variables. Given the vulnerability of this population to a changing climate, we hypothesize that climate warming could affect their body composition and, consequently, their health outcomes. To our knowledge, no prior research has examined this association. The aim of this study was to investigate the impact of climate warming on the body composition of patients undergoing MHD treatment in northern Taiwan.

## Methods

### Study area

This study was conducted in Longtan District, a suburban area of Taoyuan City, Taiwan. Taiwan, an island nation in East Asia, has a subtropical climate with hot, humid summers (30-35°C) and mild, damp winters (12-18°C). Rainfall is common year round, peaking during the summer typhoon season. Spring and fall feature moderate temperatures (20-28°C) and moderate rainfall, whereas winter often involves drizzle and light rain. To be eligible for the study, patients had to live in this area.

### Study design and patients

This longitudinal observational study was conducted from March 2018 to February 2021. In spring 2021, the study was halted because of the COVID-19 outbreak and subsequent lockdown in our facility. The inclusion criteria required patients to be 18 years or older and to have undergone 4-4.5 hours of HD treatments three times a week for at least three months. All patients received bicarbonate-based dialysis with high-flux dialyzers and standardized machine settings (blood flow 250-350 ml/min, dialysate flow 500-700 ml/min). Erythropoiesis-stimulating agents and intravenous iron sucrose were administered as needed to maintain hemoglobin levels between 10 and 11.5 g/dL 9.

Blood pressure (BP) was measured via automated monitors (IntelliVue MP5 portable patient monitor, Phillips, Netherlands) after a 10-minute rest period in the supine position. Patient dry weight was determined clinically on the basis of fluid balance assessment and body composition monitor (BCM, Fresenius Medical Care, Bad Homburg, Germany). Individuals with pacemakers, defibrillators, metallic implants, major amputations, liver cirrhosis, malignancies, or chronic inflammatory diseases were excluded because of potential interference with BCM. Seasonal data from patients hospitalized for any clinical event or hospitalized for two consecutive seasons were excluded. Ethical approval was obtained, and written informed consent was obtained from all participants.

We collected the following patients' demographic and clinical characteristics at baseline and during the study: age, sex, height, pre- and postdialysis weight, comorbidities, cause of primary renal disease, dialysis vintage, predialysis BP and interdialytic weight gain (IDWG). We calculated post-body mass index (post-BMI) as postdialysis weight (in kilograms) divided by height squared (in meters). We calculated IDWG as (predialysis weight - previous postdialysis weight)/dry weight × 100%.

### Meteorological data

The meteorological station is located in Longtan District, Taoyuan City, Taiwan. The following local meteorological data were provided by the Central Weather Bureau, Taiwan: ambient temperature, relative humidity, atmospheric pressure, and precipitation. In meteorology, seasons are defined by grouping the twelve calendar months into four 3-month periods on the basis of the annual temperature cycle. In the Northern Hemisphere, spring includes March, April, and May; summer includes June, July, and August; fall includes September, October, and November; and winter includes December, January, and February. All these measurements were taken daily and averaged for each season.

### Laboratory data

Patient blood samples were obtained immediately before the first dialysis session of the week (Monday or Tuesday) was started each month. The monthly laboratory data were calculated as the mean of the measurements for each season. The serum sodium level was corrected by increasing it by 1.6 mmol/L for every 100 mg/dL increase in the glucose level above normal 10. The normalized protein catabolic rate (nPCR) was determined on the basis of single-pool urea modeling 11.

### Body composition measurements

A trained nurse performed body composition measurements via bioimpedance spectroscopy (BCM) every month before HD treatment. Patients were positioned supine for at least 5 minutes with electrodes placed on their nonfistula forearm and ankle. The BCM device measured impedance at 50 frequencies (5 kHz to 1,000 kHz). The software automatically analyzed the data to calculate the extracellular water (ECW), intracellular water, and total body water contents via a fluid model described by Moissl *et al.* 12. Overhydration (OH), lean tissue mass, and fat tissue mass were calculated according to a physiological tissue model described by Chamney *et al.* 13. The OH value (liters) represents the difference between the patient's measured ECW and the predicted ECW under normal conditions. Relative OH (%) adjusts the OH value on the basis of the ECW. The lean tissue mass and fat tissue mass (kg) were further converted to the lean tissue index (LTI) and fat tissue index (FTI) (kg/m²) by dividing by the patient's height squared. The monthly relative OH, LTI, and FTI values were then averaged for each season throughout the study. Notably, the BCM has been extensively validated against gold standard dilution methods and dual X-ray absorptiometry 12. We perform system tests every one month using the Test Box, and suppliers perform system calibration every 24 months using the Calibration Box.

### Statistical analyses

Statistical analyses were performed via IBM SPSS Statistics version 23. Data normality was assessed with the Shapiro‒Wilk test. Descriptive statistics are presented as the means ± standard deviations for normally distributed continuous variables, medians (interquartile ranges [IQRs]) for nonparametric data, and frequencies or percentages for categorical data. Bivariate correlations between meteorological data and body composition variables were evaluated via Pearson or Spearman correlation coefficients, depending on normality. Generalized estimating equation (GEE) analyses were conducted to model longitudinal associations of repeated measures. Two GEE models were constructed for body composition analyses, adjusting for potential confounders. Model 1 included adjustments for sex, age, and dialysis vintage. Model 2 included all adjustments from Model 1 plus additional adjustments for comorbidities (cardiovascular disease and diabetes), albumin, and C-reactive protein (CRP). To investigate the potential effects of climatic variables on body composition, temperature, relative humidity, atmospheric pressure, and precipitation were added to the fully adjusted GEE models. This analysis estimated the independent influence of climatic effects on the relative OH, LTI, and FTI. A *P* value less than 0.05 was considered statistically significant.

## Results

### Meteorological data in the study area

**Figure 1:**
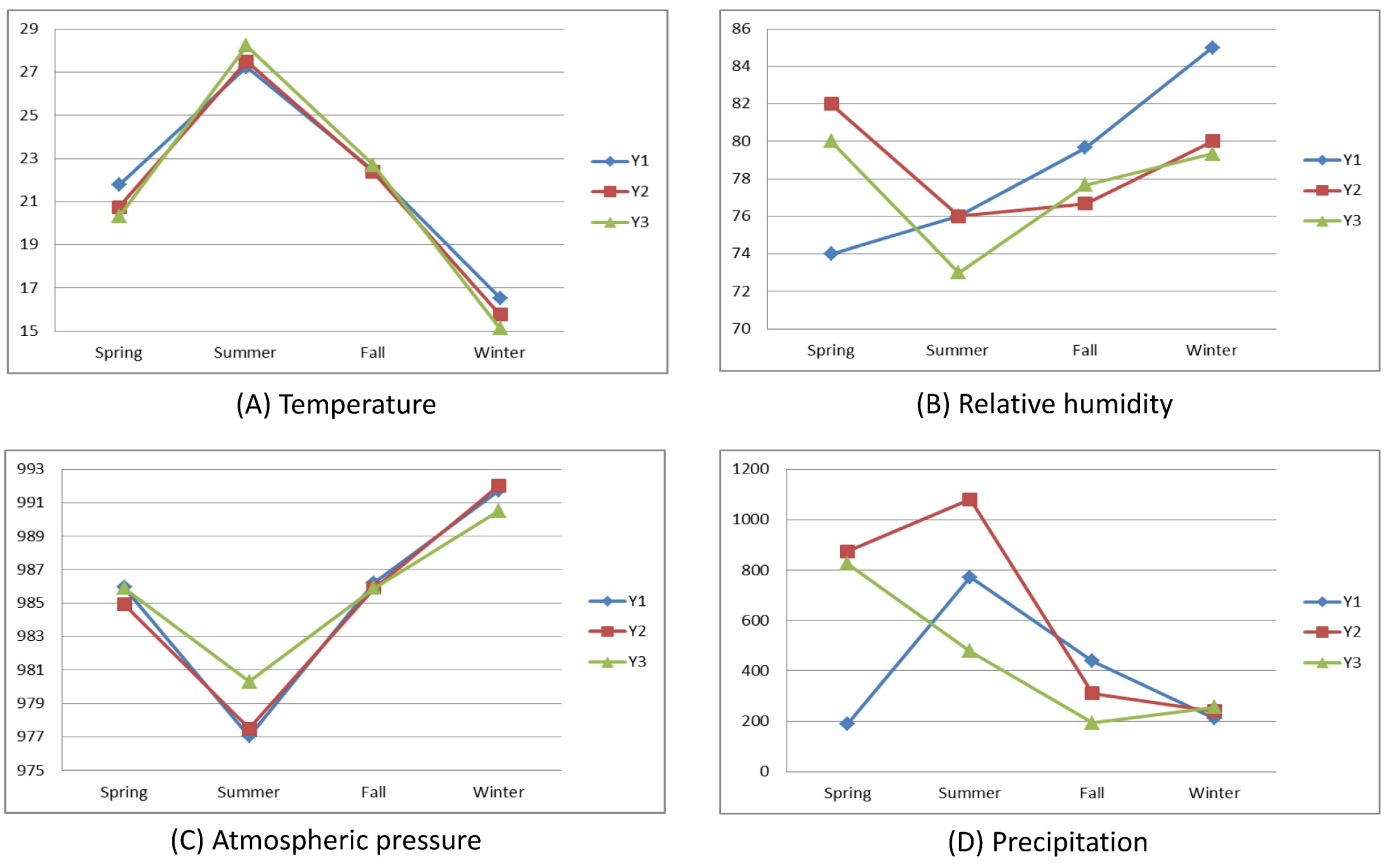
Meteorological data in the study area.

Figure 1 illustrates the mean seasonal and annual variations in the meteorological data throughout the study period. Summer presented the highest average temperatures, whereas winter presented the lowest. Notably, year three had the hottest summer, followed by years two and one. Conversely, year three also had the coldest winter, with year one having the warmest winter. Atmospheric pressure peaked in winter and dipped in summer. The variations in relative humidity and precipitation exhibited greater fluctuations.

### Patient characteristics at baseline

**Figure 2:**
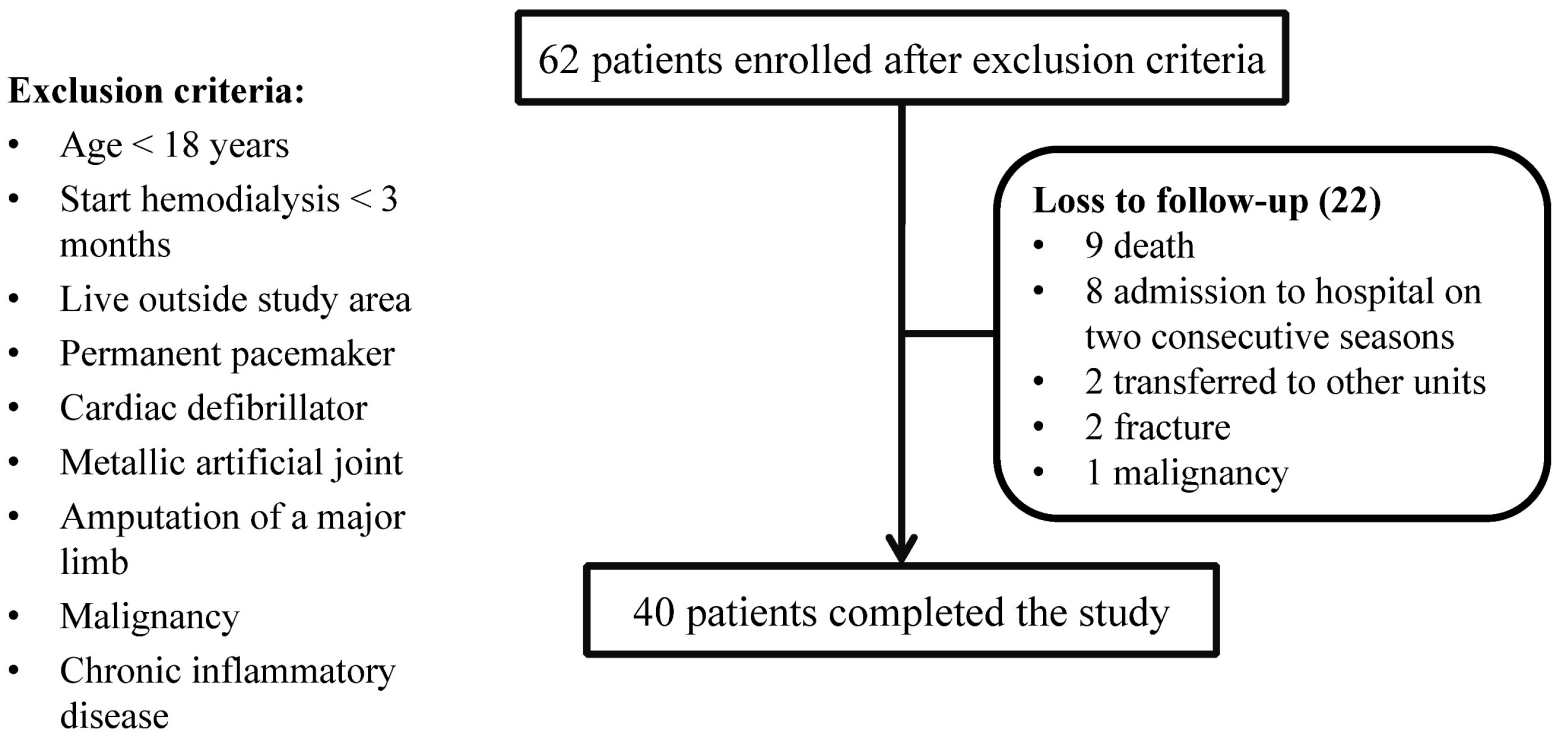
Participant flow chart.

A total of 62 patients were initially recruited in March 2018, and 40 patients completed the study. The primary reasons for withdrawal were death (9 patients, 40.9%) and admission to the hospital in two consecutive seasons (8 patients, 36.4%) (Figure 2). The demographic characteristics of those who completed the study are presented in Table 1. At baseline, the mean age was 57.9 years (range, 22-89 years); 25 (62.5%) were male; and the median dialysis vintage was 60.5 months (range, 9-333 months). The causes of primary renal disease were diabetic kidney disease (17 patients, 42.5%), chronic glomerulonephritis (12 patients, 30.0%), hypertension (9 patients, 22.5%), hereditary polycystic kidney disease (1 patient, 2.5%), and gout (1 patient, 2.5%). In terms of body composition, the mean relative OH, LTI and FTI were 11.0%, 14.2 kg/m^2^, and 10.7 kg/m^2^, respectively.

### Overall seasonal variations in BP, laboratory and body composition parameters

Table 2 shows the variations in BP and laboratory parameters for each season, with winter used as the reference. Systolic BP was significantly lower in both summer and fall than in winter. Hemoglobin, albumin, and CRP levels were not significantly different between seasons. The blood urea nitrogen (BUN), potassium, phosphate, and nPCR levels were significantly lower in the summer and fall. Conversely, sodium levels were significantly higher in summer, fall, and spring than in winter. Additionally, IDWG was significantly lower in spring, summer, and fall. Body composition also displayed seasonal trends (Table 3). Both post-BMI and FTI were lower in summer and fall than in winter. Conversely, the relative OH was greater in spring, summer, and fall. The LTI was initially lower in spring; however, summer emerged as the only season with significantly higher LTI after adjusting for sex (*P* < .001), age (*P* < .001) and dialysis vintage (*P* > 0.05). According to the fully adjusted models, the findings for post-BMI, relative OH and FTI were significant and consistent.

### Annual variations in BP, laboratory and body composition parameters according to seasonality

Compared with year one as a baseline, our analysis revealed some annual variations in the study parameters across each season. BP and most laboratory values remained stable throughout the year, except for the sodium and potassium levels (Table 4, S1-3). Sodium levels were significantly higher in the spring of year three and throughout the summer, fall, and winter of years two and three. Conversely, potassium levels decreased in the summer of year three. The IDWG also decreased significantly during summer, fall, and winter of year three. The post-BMI showed no significant annual variation except for a single observation with a lower level in the winter of year three (Table 5, S4-6). However, this difference became statistically insignificant after adjusting for sex (*P* < 0.001), age (*P* < 0.001) and dialysis vintage (*P* > 0.05). Notably, relative OH levels were consistently higher in spring, summer, and fall of year three across all the analysis models. Finally, the LTI was lower while the FTI was greater in the summers of years two and three, and this pattern held true even after adjusting for all relevant factors.

### Associations of meteorological data with body composition

The bivariate correlations between the meteorological data and body composition parameters examined in the present study are presented in Table 6. The relative OH was directly correlated with the ambient temperature. The FTI was directly correlated with atmospheric pressure and inversely correlated with ambient temperature. We found no significant correlation between meteorological data and post-BMI or LTI. However, when meteorological variables were added to the fully adjusted GEE models, both warmer temperature and higher atmospheric pressure emerged as significant positive factors for relative OH (Table 7). LTI exhibited a positive association with humidity, whereas all other climatic variables (temperature, pressure, and precipitation) had an inverse effect. Finally, the FTI demonstrated a positive correlation with all the climatic variables we examined in the adjusted models, except for relative humidity.

## Discussion

This study was conducted to examine the impact of rising ambient temperatures on body composition in an ESRD population of MHD residents on a subtropical island. Over the three-year study period, summer and fall had higher temperatures than did winter, with year three experiencing the highest temperatures. The major findings were that relative OH was greater in spring, summer, and fall than in winter, and these levels were further elevated in year three compared with year one. The LTI was highest in summer but showed a declining trend over the years, whereas the FTI was lowest in summer and fall but increased throughout the study period. The relative OH and FTI were directly correlated, and the LTI was inversely correlated with temperature, suggesting that climate warming may contribute to fluid overload, fat gain and muscle loss in MHD patients. Furthermore, the study revealed seasonal and annual variations in BP, IDWG, and laboratory data among MHD patients.

Among seasonal variations, fluctuations in BP, temperature, laboratory values, and mortality in ESRD patients undergoing HD have been reported 8, 14-19. Consistent with observations in the general population, several studies have identified a distinct seasonal pattern in predialysis BP, with a peak in winter and a nadir in summer 14-16, 18. Our findings align with these prior studies, which demonstrated significantly lower predialysis systolic BP during the summer and fall months. Seasonal variations in ambient temperature, leading to potential vasoconstriction and vasodilation, along with changes in diet and physical activity patterns, could explain this finding 20.

Laboratory parameters, including hemoglobin, albumin and CRP, also undergo cyclic changes in HD patients; however, the seasonal patterns vary across different climates and studies 15, 17-19, 21, 22. In our study, no seasonal variations were observed in the serum albumin or CRP levels, possibly due to the exclusion of patient data collected during periods of acute clinical events. Predialysis BUN, potassium, phosphate and nPCR levels were significantly lower in the summer and fall than in the other months, corroborating the findings of previous studies 16, 17, 19. Ambient temperature can influence appetite, with hot weather decreasing energy intake and cold weather increasing it 23, 24. A systematic review and meta-analysis revealed that energy intake tended to be greater in winter and spring and lower in summer 25. Since summer and fall are hot seasons in Taiwan, the observed lower BUN, potassium, phosphate, and nPCR levels in these seasons suggest that food intake is diminished due to seasonal effects.

In the general population, previous studies reported that body weight and BMI were lower in warmer seasons and higher in colder seasons 26, 27. In HD patients, body mass and body composition also vary between seasons. Two studies reported that body weight and BMI in HD patients were lowest in hot months and highest in cold months 21, 28. Another study conducted by Broers *et al.* reported that post-HD weight was lower in spring and summer 8. Contrary to these findings, Ilic Begovic *et al.* reported no significant differences in BMI between seasons 22. Our study revealed that there was seasonal variation in post-HD BMI, which was lower in summer and fall. BUN, potassium, phosphate and nPCR were lower in summer and fall, suggesting reduced food intake in these two seasons and subsequently decreased BMI 25.

Several studies have shown seasonal variations in body composition. Broers *et al.* reported that lean tissue mass was highest in summer and that fat mass was highest in winter 8. Similarly, Ilic Begovic *et al.* reported a seasonal pattern in which the FTI was highest in winter and lowest in summer, whereas the LTI was highest in summer and lowest in winter 22. Our findings corroborate these observations, with the LTI being greater and the FTI being lower in summer and fall than in winter. The seasonal variation in fat tissue mass is well documented in the general population. A previous study demonstrated lower body fat percentages during hot seasons across three populations from different climate areas 29. This trend in our study might be explained by potentially lower energy intake and higher physical activity-induced energy expenditure in summer and fall 30. While research on seasonal LTI variations in healthy individuals is limited, Broers *et al.* linked higher summer LTI to lower inflammation in MHD patients 8. However, neither our study nor Ilic Begovic *et al.* reported this association. Ilic Begovic *et al.* reported that oxidative stress is correlated with fat tissue and may have a negative influence on lean tissue 22. Alternatively, increased physical activity during summer might contribute to a significant decrease in FTI and increase in LTI 30. Since seasonal variation in body mass is related mainly to changes in fat mass, these findings also explain the seasonal variation in post-BMI 30.

Our findings indicate higher relative OH in spring, summer, and fall than in winter. This aligns with studies by Broers *et al.*, who reported higher ECW and fluid overload during warmer months 8. Similarly, Ilic Begovic *et al.* reported a seasonal pattern with higher OH in summer 22. Some factors might explain these seasonal variations. Higher ambient temperatures in spring, summer, and fall could lead to lower food intake, potentially causing a decrease in fat mass and a relative increase in fluid overload. Additionally, while lean tissue mass might increase, the decrease in fat mass may be clinically insignificant, further contributing to increased OH. Interestingly, despite higher OH in summer than in winter, BP and IDWG were lower, and serum sodium was higher during the warmer seasons. This suggests that increased perspiration and insensible fluid loss due to higher temperatures may outweigh the rise in fluid intake driven by thirst, leading to lower IDWG. This finding aligns with previous research demonstrating lower IDWG in hot months 8, 28. Our prior study with MHD patients demonstrated that IDWG but not fluid overload was inversely associated with predialysis serum sodium levels, supporting our findings in this study 31. While fluid status significantly impacts BP in MHD patients, our study, similar to previous research, suggests a stronger association between seasonal variations in BP and IDWG compared to chronic fluid overload 32.

While we observed seasonal variations in BP and some laboratory parameters, the influence of climate warming was limited to specific parameters in MHD patients. While BP was lower in warmer seasons, a large Chinese study reported small decreases in systolic BP with larger temperature increases (0.74 mmHg overall and 1.62 mmHg in the warm season of the subtropical zone) 33. This suggests that the seasonal temperature changes (less than 1.5°C) in our study may not have been large enough to produce statistically significant BP changes. Similarly, annual variations in hemoglobin, BUN, phosphate, albumin, nPCR, and CRP levels were not observed with climate warming. However, the sodium and potassium levels did change. Notably, decreased summer potassium levels across the study period suggest that climate warming may have led to a slight reduction in dietary potassium intake.

Our study suggests a link between climate warming and changes in body composition. Seasonal variations previously revealed a lower post-BMI and FTI with a higher LTI in summer. However, during our three-year study, the summer post-BMI remained stable, while the FTI increased and the LTI decreased with increasing temperature; this aligns with growing evidence suggesting that climate warming may contribute to human obesity, as increased FTI reflects rising ambient temperature. In addition to temperature, factors such as relative humidity, pressure, and precipitation also influence LTI/FTI changes. While physical activity often increases during hot weather, research suggests that this correlation may reverse when temperatures exceed a certain threshold 34. Given Taiwan's hot climate, rising summer temperatures throughout the study may have led to decreased physical activity and lower energy expenditure; this aligns with the established link between sedentary lifestyles—including increased screen time, unhealthy eating, and overconsumption—and the prevalence of obesity 35. The observed association between precipitation and increased FTI suggests a decrease in activity during rainy periods, further promoting a sedentary lifestyle 36. The underlying mechanisms behind these climate-related changes remain unclear. Climate extremes may act as stressors, triggering physiological changes such as upregulated inflammatory factors, intestinal injury, and the generation of reactive oxygen species, which are believed to affect cells throughout the body 37, 38. Additionally, as previously reported, excessive fat tissue can gradually increase oxidative stress, leading to muscle loss 22, 39. This combination of increased body fat and muscle wasting, known as sarcopenic obesity, is linked to increased hospitalization and mortality risks in MHD patients 40.

This study revealed that relative OH levels increased during summer and fall across the three years, which aligns with the observations of seasonal variations where OH levels were relatively high during hot months. This positive correlation between relative OH and ambient temperature suggests a novel link between climate warming and fluid overload in MHD patients. Interestingly, a decrease in IDWG during summer and fall, despite rising temperatures, argues against increased fluid intake as a cause of overload. Additionally, the lack of annual variation in CRP levels makes fluid overload due to inflammation less likely. The stable post-BMI across the year suggests that fluid retention might mask a decrease in muscle mass exceeding any increase in fat mass. Notably, the serum sodium levels increased each season across the study years. This trend mirrored the inverse seasonal changes in IDWG, suggesting that the negative association between sodium and IDWG holds true even under climate warming conditions 31. While seasonal variations showed synchronous changes between BP and IDWG, there was no significant association between BP and IDWG or chronic fluid overload in the context of climate warming. This finding highlights the complex interplay between temperature, fluid status, vasodilation/constriction, and other factors.

This study also considered atmospheric pressure as a potential factor influencing body composition. Research has shown significant reductions in body weight, fat mass, muscle mass, and/or body water at high altitudes (>1500 m) due to lower pressure-induced hypoxia 41, 42. However, the pressure changes in this study were much smaller (approximately 20 hPa), limiting any hypoxic effects. High-pressure systems can lead to stable, clear weather conditions that may intensify heat waves in certain areas, thereby exacerbating the impacts of rising temperatures on body composition in MHD patients.

Our study offers a unique perspective on the impact of climate warming on body composition through its longitudinal design, allowing us to account for time-varying factors that might influence the outcome. Additionally, we are the first to explore this association in MHD patients. However, some limitations require consideration. The modest sample size necessitates further research with larger cohorts for broader applicability. Furthermore, excluding patients with certain medical devices may have introduced selection bias, potentially creating a healthier study population. While we relied on outdoor meteorological data, which aligns with previous research demonstrating a connection between indoor and outdoor conditions, it might not fully capture the participants' true indoor environment 43, 44. Finally, dietary intake and physical activity, both of which are known to influence body composition, were not factored into our analysis. The Taiwanese diet, often characterized by salty and oily content, and the prevalence of night markets in Taiwanese food culture, could contribute to body composition changes in MHD patients in Taiwan. While our study did not explicitly address these factors, our findings may provide insights into the potential effects of climate warming on body composition in other populations, especially those with similar food culture or lifestyle. Expanding our research to include populations from diverse climates and regions would help to enhance the generalizability of our findings.

Regular assessment of body composition is essential for optimizing the care of MHD patients in a changing climate. Common assessment methods include anthropometric measurements, bioimpedance analysis, and dual-energy X-ray absorptiometry. By tracking changes in muscle mass, fat mass, and fluid balance, healthcare providers can identify and address potential effects of climate warming on body composition. Based on these observations, physicians can adjust dialysis treatment plans, target dry weight, or provide guidance on hydration, nutrition, and lifestyle modifications. Pharmacological interventions, such as glucagon-like peptide-1 agonists, have shown promise in inducing weight loss by reducing fat mass while preserving skeletal muscle mass 45. Future research should focus on developing glucagon-like peptide-1-based and other therapies aimed at improving muscle mass, composition, and strength in MHD patients, helping them adapt to the challenges of climate warming.

## Conclusions

Our study highlights the potential impact of climate warming on body composition in MHD patients residing in subtropical regions. Given this population's pre-existing vulnerabilities in fluid balance and cardiovascular health, these changes are particularly concerning. Further research is warranted to explore these effects in geographically diverse areas with different climates, which will be crucial to ensure optimal care for dialysis patients in a changing climate.

## Supplementary Material

Supplementary tables.

## Figures and Tables

**Table 1:** Baseline characteristics of the study participants

Parameters	Data
Gender (% male)	62.5
Age (years)	57.9 ± 13.7
Cardiovascular disease (%)	57.5
Diabetes mellitus (%)	42.5
Dialysis vintage (months)	60.5 (28.3-123.0)*
Systolic BP (mmHg)	159.9 ± 23.2
Hemoglobin (g/dL)	10.7 ± 1.2
BUN (mg/dL)	65.0 ± 13.4
Creatinine (mg/dL)	12.3 ± 2.6
Sodium (mmol/L)	135.1 ± 2.6
Potassium (mmol/L)	4.5 ± 0.7
Phosphate (mg/dL)	5.3 ± 1.1
Albumin (g/dL)	3.8 ± 0.3
CRP (mg/dL)	0.8 ± 0.7
nPCR (g/kg/day)	1.1 ± 0.2
Post BMI (kg/m^2^)	24.6 ± 3.9
IDWG (%)	4.6 ± 1.6
Relative OH (%)	11.0 ± 6.6
LTI (kg/m^2^)	14.2 ± 3.4
FTI (kg/m^2^)	10.7 ± 4.1

BMI, body mass index; BP, blood pressure; BUN, blood urea nitrogen; CRP, C-reactive protein; FTI, fat tissue index; IDWG, interdialytic weight gain; LTI, lean tissue index; nPCR, normalized protein catabolic rate; OH, overhydration *Expressed as the median (interquartile range)

**Table 2:** Overall seasonal variations in parameters over the three-year study period, with winter as a reference

Parameters	Season	B	95% CI	Wald χ^2^	*P*
Systolic BP	Spring	-5.73	-12.17-0.72	3.03	>0.05
	Summer	-9.23	-14.33- -4.13	12.60	<0.001
	Fall	-5.55	-10.21- -0.90	5.48	0.019
Hemoglobin	Spring	-0.17	-0.48-0.14	1.20	>0.05
	Summer	-0.18	-0.49-0.14	1.24	>0.05
	Fall	-0.15	-0.36-0.06	1.95	>0.05
BUN	Spring	1.51	-0.72-3.75	1.76	>0.05
	Summer	-4.30	-6.89- -1.71	10.61	0.001
	Fall	-5.04	-7.33- -2.75	18.55	<0.001
Sodium	Spring	1.35	0.87-1.83	30.15	<0.001
	Summer	1.60	1.02-2.17	29.44	<0.001
	Fall	1.30	0.87-1.73	35.34	<0.001
Potassium	Spring	-0.04	-0.15-0.06	0.67	>0.05
	Summer	-0.22	-0.33- -0.11	14.35	<0.001
	Fall	-0.27	-0.36- -0.19	38.13	<0.001
Phosphate	Spring	-0.18	-0.42-0.06	2.28	>0.05
	Summer	-0.46	-0.73- -0.19	11.30	0.001
	Fall	-0.31	-0.51- -0.11	9.22	0.002
Albumin	Spring	-0.03	-0.11-0.05	0.44	>0.05
	Summer	-0.05	-0.13-0.03	1.38	>0.05
	Fall	-0.01	-0.06-0.04	0.13	>0.05
CRP	Spring	-0.02	-0.16-0.12	0.07	>0.05
	Summer	-0.08	-0.25-0.10	0.77	>0.05
	Fall	0.00	-0.14-0.15	0.00	>0.05
nPCR	Spring	-0.00	-0.04-0.03	0.01	>0.05
	Summer	-0.06	-0.10- -0.02	10.14	0.001
	Fall	-0.08	-0.11- -0.05	22.31	<0.001
IDWG	Spring	-0.53	-0.89- -0.18	8.64	0.003
	Summer	-1.11	-1.38- -0.83	62.98	<0.001
	Fall	-0.66	-0.91- -0.40	25.85	<0.001

BP, blood pressure; BUN, blood urea nitrogen; CRP, C-reactive protein; IDWG, interdialytic weight gain; nPCR, normalized protein catabolic rate

**Table 3:** Overall seasonal variations in body composition over the three-year study period, with winter as a reference

Parameters	Models	Season	B	95% CI	Wald χ^2^	*P*
Post-BMI	Unadjusted	Spring	-0.67	-1.80-0.47	1.32	>0.05
		Summer	-1.08	-1.91- -0.25	6.52	0.011
		Fall	-0.79	-1.20- -0.39	14.61	<0.001
	Model 1	Spring	0.09	-0.54-0.73	0.08	>0.05
		Summer	-0.57	-1.05- -0.08	5.23	0.022
		Fall	-0.53	-0.82- -0.25	13.84	<0.001
	Model 2	Spring	0.12	-0.52-0.75	0.13	>0.05
		Summer	-0.51	-0.98- -0.05	4.63	0.031
		Fall	-0.52	-0.78- -0.27	16.06	<0.001
Relative OH	Unadjusted	Spring	3.76	1.55-5.97	11.10	0.001
		Summer	3.54	1.52-5.56	11.74	0.001
		Fall	2.08	1.02-3.14	14.69	<0.001
	Model 1	Spring	3.17	1.34-4.99	11.57	0.001
		Summer	3.14	1.30-4.97	11.26	0.001
		Fall	1.86	0.85-2.86	13.19	<0.001
	Model 2	Spring	1.98	0.84-3.11	11.63	0.001
		Summer	2.19	0.90-3.47	11.14	0.001
		Fall	1.59	0.79-2.38	15.24	<0.001
LTI	Unadjusted	Spring	-1.00	-1.70- -0.31	7.95	0.005
		Summer	0.16	-0.33-0.63	0.40	>0.05
		Fall	-0.03	-0.31-0.24	0.06	>0.05
	Model 1	Spring	-0.27	-0.74-0.19	1.33	>0.05
		Summer	0.64	0.32-0.97	15.37	<0.001
		Fall	0.21	-0.01-0.43	3.50	>0.05
	Model 2	Spring	-0.26	-0.72-0.19	1.29	>0.05
		Summer	0.67	0.36-0.98	17.73	<0.001
		Fall	0.21	-0.00-0.43	3.78	>0.05
FTI	Unadjusted	Spring	0.28	-0.97-1.52	0.19	>0.05
		Summer	-1.26	-2.09- -0.43	8.88	0.003
		Fall	-0.76	-1.14- -0.38	15.29	<0.001
	Model 1	Spring	0.36	-0.56-1.27	0.58	>0.05
		Summer	-1.21	-1.79- -0.62	16.38	<0.001
		Fall	-0.73	-1.01- -0.45	25.46	<0.001
	Model 2	Spring	0.34	-0.60-1.29	0.51	>0.05
		Summer	-1.19	-1.78- -0.61	15.98	<0.001
		Fall	-0.73	-1.00- -0.46	28.23	<0.001

BMI, body mass index; FTI, fat tissue index; LTI, lean tissue index; OH, overhydration

**Table 4:** Annual variations in parameters in summer, with year one as a reference

Parameter/Model	Year	B	95% CI	Wald χ^2^	*P*
Systolic BP	Year three	-1.05	-6.12-4.03	0.16	>0.05
	Year two	-1.27	-7.17-4.64	0.18	>0.05
Hemoglobin	Year three	-0.07	-0.45-0.32	0.12	>0.05
	Year two	-0.05	-0.36-0.27	0.09	>0.05
BUN	Year three	0.54	-4.33-5.42	0.05	>0.05
	Year two	2.21	-3.38-7.80	0.60	>0.05
Sodium	Year three	1.81	1.16-2.46	29.72	<0.001
	Year two	0.80	0.13-1.46	5.55	0.019
Potassium	Year three	-0.22	-0.39- -0.04	5.85	0.016
	Year two	0.08	-0.09-0.25	0.94	>0.05
Phosphate	Year three	0.13	-0.21-0.46	0.56	>0.05
	Year two	0.06	-0.17-0.29	0.30	>0.05
Albumin	Year three	0.06	-0.00-0.13	3.35	>0.05
	Year two	0.03	-0.01-0.06	2.22	>0.05
CRP	Year three	0.08	-0.07-0.22	1.17	>0.05
	Year two	0.08	-0.12-0.28	0.60	>0.05
nPCR	Year three	0.06	-0.02-0.15	1.96	>0.05
	Year two	0.07	-0.01-0.15	2.74	>0.05
IDWG	Year three	-1.42	-1.88- -0.95	35.76	<0.001
	Year two	-0.37	-0.76-0.03	3.37	>0.05

BP, blood pressure; BUN, blood urea nitrogen; BP, blood pressure; CRP, C-reactive protein; IDWG, interdialytic weight gain; nPCR, normalized protein catabolic rate

**Table 5:** Annual variations in body composition in summer, with the first year used as a reference

Parameters	Models	Year	B	95% CI	Wald χ^2^	*P*
Post-BMI	Unadjusted	Year three	-0.17	-0.71-0.36	0.41	>0.05
		Year two	0.16	-0.24-0.56	0.64	>0.05
Model 1	Model 1	Year three	0.15	-0.52-0.82	0.19	>0.05
		Year two	0.32	-0.13-0.78	1.96	>0.05
Model 2	Model 2	Year three	-0.11	-0.83-0.61	0.09	>0.05
		Year two	0.21	-0.25-0.66	0.77	>0.05
Relative OH	Unadjusted	Year three	2.04	0.15-3.93	4.45	0.035
		Year two	-0.04	-1.46-1.39	0.00	>0.05
Model 1	Model 1	Year three	1.88	0.12-3.64	4.41	0.036
		Year two	-0.11	-1.54-1.32	0.02	>0.05
Model 2	Model 2	Year three	3.44	1.70-5.17	15.08	<0.001
		Year two	0.62	-0.75-1.98	0.79	>0.05
LTI	Unadjusted	Year three	-1.14	-1.50- -0.79	39.94	<0.001
		Year two	-1.20	-1.48- -0.92	69.61	<0.001
Model 1	Model 1	Year three	-1.02	-1.46- -0.57	20.13	<0.001
		Year two	-1.14	-1.46- -0.81	47.60	<0.001
Model 2	Model 2	Year three	-1.15	-1.61- -0.69	23.76	<0.001
		Year two	-1.19	-1.50- -0.88	56.66	<0.001
FTI	Unadjusted	Year three	0.94	0.30-1.59	8.14	0.004
		Year two	1.35	0.88-1.81	31.98	<0.001
Model 1	Model 1	Year three	1.16	0.35-1.98	7.76	0.005
		Year two	1.46	0.91-2.01	26.78	<0.001
Model 2	Model 2	Year three	1.00	0.09-1.92	4.63	0.031
		Year two	1.38	0.80-1.96	21.75	<0.001

BMI, body mass index; FTI, fat tissue index; LTI, lean tissue index; OH, overhydration

**Table 6:** Bivariate correlations of meteorological parameters with body composition parameters in the study population

Meteorological parameters	Body composition	*r*	*P*	Bootstripping analysis		
				bias	SD	95% CI
Temperature	Post-BMI	-0.05	>0.05	0.000	0.05	-0.14-0.04
	Relative OH	0.10	0.040	0.000	0.05	0.00-0.18
	LTI	0.07	>0.05	0.000	0.05	-0.02-0.17
	FTI	-0.10	0.029	0.000	0.05	-0.19- -0.01
Relative humidity	Post-BMI	0.04	>0.05	0.001	0.05	-0.05-0.13
	Relative OH	-0.09	>0.05	-0.001	0.05	-0.18-0.00
	LTI	-0.05	>0.05	-0.001	0.04	-0.14-0.03
	FTI	0.08	>0.05	0.001	0.04	-0.01-0.17
Atmospheric pressure	Post-BMI	0.03	>0.05	0.002	0.05	-0.06-0.12
	Relative OH	-0.06	>0.05	-0.001	0.05	-0.16-0.03
	LTI	-0.08	>0.05	0.001	0.05	-0.18-0.00
	FTI	0.10	0.028	0.000	0.05	0.01-0.19
Precipitation	Post-BMI	-0.01	>0.05	-0.002	0.05	-0.10-0.08
	Relative OH	0.01	>0.05	0.001	0.05	-0.08-0.10
	LTI	0.04	>0.05	-0.002	0.05	-0.05-0.13
	FTI	-0.04	>0.05	0.000	0.05	-0.13-0.05

BMI, body mass index; FTI, fat tissue index; LTI, lean tissue index; OH, overhydration

**Table 7:** Associations of meteorological parameters with body composition parameters in GEE models

Body composition	Meteorological parameters	Model 1		Model 2	
		B	*P*	B	*P*
Post-BMI	Year one	0.095	>0.05	0.071	>0.05
	Year two	0.201	>0.05	0.135	>0.05
	Temperature			-0.018	>0.05
	Relative humidity			-0.012	>0.05
	Atmospheric pressure			0.047	>0.05
	Precipitation			0.000	>0.05
Relative OH	Year one	-2.253	0.001	-1.884	0.006
	Year two	-1.903	<.001	-1.618	<0.001
	Temperature			0.302	0.016
	Relative humidity			-0.099	>0.05
	Atmospheric pressure			0.276	0.022
	Precipitation			0.001	>0.05
LTI	Year one	0.247	>0.05	0.133	>0.05
	Year two	-0.121	>0.05	-0.046	>0.05
	Temperature			-0.105	<0.001
	Relative humidity			0.040	0.049
	Atmospheric pressure			-0.203	<0.001
	Precipitation			-0.001	<0.001
FTI	Year one	-0.146	>0.05	-0.036	>0.05
	Year two	0.330	>0.05	0.190	>0.05
	Temperature			0.093	0.016
	Relative humidity			-0.055	>0.05
	Atmospheric pressure			0.257	<0.001
	Precipitation			0.002	<0.001

BMI, body mass index; FTI, fat tissue index; LTI, lean tissue index; OH, overhydration

## Abbreviations

BCM: body composition monitor
BMI: body mass index
BP: blood pressure
BUN: blood urea nitrogen
CRP: C-reactive protein
ECW: extracellular water
ESRD: end-stage renal disease
FTI: fat tissue index
GEE: generalized estimating equation
IDWG: interdialytic weight gain
LTI: lean tissue index
MHD: maintenance hemodialysis
nPCR: normalized protein catabolic rate
OH: overhydration
