# Supplementary material for: Impacts of Climate Warming on the Body Composition of Patients Undergoing Maintenance Hemodialysis
Source: Int J Med Sci. 2024 Oct 21;21(14):2759–69. doi: 10.7150/ijms.101232 (PMC11539392; doi:10.7150/ijms.101232)
Supplement: Supplementary file 1 — Supplementary tables. [file ijmsv21p2759s1.pdf]

**Table S1 Annual variations in parameters in spring, with year one as a reference**

| **Parameters** | **Year** | **B** | **95% CI** | **Wald $\chi^2$** | ***P*** |
|---|---|---|---|---|---|
| Systolic BP | Year three | 0.79 | −7.90−9.48 | 0.03 | >0.05 |
| | Year two | −0.04 | −5.48−5.41 | 0.00 | >0.05 |
| Hemoglobin | Year three | −0.15 | −0.46−0.16 | 0.93 | >0.05 |
| | Year two | −0.07 | −0.37−0.23 | 0.22 | >0.05 |
| BUN | Year three | 1.66 | −2.96−6.28 | 0.50 | >0.05 |
| | Year two | −0.60 | −4.84−3.65 | 0.08 | >0.05 |
| Sodium | Year three | 1.28 | 0.70−1.86 | 18.74 | <0.001 |
| | Year two | 0.25 | −0.33−0.82 | 0.71 | >0.05 |
| Potassium | Year three | 0.11 | −0.11−0.33 | 0.98 | >0.05 |
| | Year two | 0.14 | −0.06−0.33 | 1.92 | >0.05 |
| Phosphate | Year three | 0.03 | −0.29−0.35 | 0.03 | >0.05 |
| | Year two | 0.18 | −0.14−0.49 | 1.18 | >0.05 |
| Albumin | Year three | 0.03 | −0.01−0.07 | 2.56 | >0.05 |
| | Year two | 0.02 | −0.01−0.06 | 1.51 | >0.05 |
| CRP | Year three | 0.11 | −0.06−0.28 | 1.60 | >0.05 |
| | Year two | 0.07 | −0.11−0.26 | 0.59 | >0.05 |
| nPCR | Year three | −0.04 | −0.08−0.00 | 3.17 | >0.05 |
| | Year two | −0.07 | −0.14−0.01 | 2.91 | >0.05 |
| IDWG | Year three | −0.27 | −0.61−0.07 | 2.38 | >0.05 |
| | Year two | −0.11 | −0.43−0.21 | 0.45 | >0.05 |

BP, blood pressure; BUN, blood urea nitrogen; BP, blood pressure; CRP, C-reactive protein; IDWG, interdialytic weight gain; nPCR, normalized protein catabolic rate

**Table S2 Annual variations in parameters in fall, with year one as a reference**

| Parameters | Year | B | 95% CI | Wald $\chi^2$ | *P* |
|---|---|---|---|---|---|
| Systolic BP | Year three | −2.82 | −10.01−4.37 | 0.59 | >0.05 |
| | Year two | −2.92 | −8.61−2.77 | 1.01 | >0.05 |
| Hemoglobin | Year three | −0.05 | −0.36−0.27 | 0.08 | >0.05 |
| | Year two | −0.17 | −0.43−0.09 | 1.65 | >0.05 |
| BUN | Year three | 1.96 | −3.63−7.55 | 0.47 | >0.05 |
| | Year two | 2.53 | −1.03−6.09 | 1.95 | >0.05 |
| Sodium | Year three | 1.53 | 0.83−2.24 | 18.13 | <0.001 |
| | Year two | 1.32 | 0.77−1.87 | 22.44 | <0.001 |
| Potassium | Year three | 0.04 | −0.13−0.21 | 0.23 | >0.05 |
| | Year two | 0.09 | −0.04−0.22 | 1.78 | >0.05 |
| Phosphate | Year three | −0.17 | −0.53−0.20 | 0.80 | >0.05 |
| | Year two | 0.08 | −0.19−0.35 | 0.30 | >0.05 |
| Albumin | Year three | −0.01 | −0.08−0.05 | 0.18 | >0.05 |
| | Year two | −0.02 | −0.05−0.01 | 2.39 | >0.05 |
| CRP | Year three | 0.01 | −0.25−0.28 | 0.01 | >0.05 |
| | Year two | 0.03 | −0.18−0.25 | 0.09 | >0.05 |
| nPCR | Year three | 0.00 | −0.05− 0.06 | 0.02 | >0.05 |
| | Year two | 0.03 | −0.04−0.09 | 0.74 | >0.05 |
| IDWG | Year three | −1.02 | −1.47− −0.57 | 19.67 | <0.001 |
| | Year two | 0.00 | −0.43−0.43 | 0.00 | >0.05 |

BP, blood pressure; BUN, blood urea nitrogen; BP, blood pressure; CRP, C-reactive protein; IDWG, interdialytic weight gain; nPCR, normalized protein catabolic rate

**Table S3 Annual variations in parameters in winter, with year one as a reference**

| Parameters | Year | B | 95% CI | Wald $\chi^2$ | *p* |
|---|---|---|---|---|---|
| Systolic BP | Year three | 0.25 | −11.51−12.00 | 0.00 | >0.05 |
| | Year two | 1.17 | −5.45−7.79 | 0.12 | >0.05 |
| Hemoglobin | Year three | 0.10 | −0.24−0.44 | 0.32 | >0.05 |
| | Year two | −0.07 | −0.39−0.26 | 0.16 | >0.05 |
| BUN | Year three | 0.09 | −4.38−4.56 | 0.00 | >0.05 |
| | Year two | −0.70 | −4.58−3.18 | 0.13 | >0.05 |
| Sodium | Year three | 2.88 | 1.96−3.80 | 37.68 | <0.001 |
| | Year two | 2.83 | 2.25−3.40 | 92.97 | <0.001 |
| Potassium | Year three | −0.16 | −0.34−0.01 | 3.46 | >0.05 |
| | Year two | 0.01 | −0.20−0.21 | 0.00 | >0.05 |
| Phosphate | Year three | −0.01 | −0.35−0.34 | 0.00 | >0.05 |
| | Year two | 0.01 | −0.34−0.36 | 0.00 | >0.05 |
| Albumin | Year three | 0.04 | −0.03−0.10 | 1.09 | >0.05 |
| | Year two | 0.01 | −0.03−0.05 | 0.13 | >0.05 |
| CRP | Year three | 0.08 | −0.11−0.27 | 0.68 | >0.05 |
| | Year two | 0.09 | −0.08−0.27 | 1.18 | >0.05 |
| nPCR | Year three | 0.02 | −0.05−0.09 | 0.34 | >0.05 |
| | Year two | 0.05 | −0.01−0.10 | 2.44 | >0.05 |
| IDWG | Year three | −1.00 | −1.55− −0.46 | 13.03 | <0.001 |
| | Year two | −0.09 | −0.51−0.33 | 0.18 | >0.05 |

BP, blood pressure; BUN, blood urea nitrogen; BP, blood pressure; CRP, C-reactive protein; IDWG, interdialytic weight gain; nPCR, normalized protein catabolic rate

**Table S4 Annual variations in body composition in spring, with year one as a reference**

| Parameters | Year | B | 95% CI | Wald $\chi^2$ | $p$ |
|---|---|---|---|---|---|
| Post-BMI | Year three | −0.20 | −0.43−0.03 | 2.82 | >0.05 |
| | Year two | −0.03 | −0.18−0.12 | 0.16 | >0.05 |
| Model 1 | Year three | 0.06 | −0.41−0.53 | 0.07 | >0.05 |
| | Year two | 0.10 | −0.14−0.34 | 0.65 | >0.05 |
| Model 2 | Year three | 0.04 | −0.42−0.51 | 0.03 | >0.05 |
| | Year two | 0.09 | −0.17−0.34 | 0.44 | >0.05 |
| Relative OH | Year three | 1.91 | 0.63−3.19 | 8.58 | 0.003 |
| | Year two | −0.77 | −1.74−0.20 | 2.42 | >0.05 |
| Model 1 | Year three | 1.79 | 0.40−3.19 | 6.33 | 0.012 |
| | Year two | −0.82 | −1.83−0.18 | 2.59 | >0.05 |
| Model 2 | Year three | 1.80 | 0.21−3.39 | 4.94 | 0.026 |
| | Year two | −0.80 | −1.90−0.30 | 2.01 | >0.05 |
| LTI | Year three | 0.08 | −0.22−0.39 | 0.29 | >0.05 |
| | Year two | −0.15 | −0.41−0.11 | 1.22 | >0.05 |
| Model 1 | Year three | 0.22 | −0.19−0.63 | 1.10 | >0.05 |
| | Year two | −0.08 | −0.37−0.21 | 0.28 | >0.05 |
| Model 2 | Year three | 0.16 | −0.27−0.59 | 0.53 | >0.05 |
| | Year two | −0.12 | −0.43−0.19 | 0.58 | >0.05 |
| FTI | Year three | −0.30 | −0.60−0.01 | 3.71 | >0.05 |
| | Year two | 0.12 | −0.16−0.40 | 0.68 | >0.05 |
| Model 1 | Year three | −0.15 | −0.68−0.37 | 0.32 | >0.05 |
| | Year two | 0.19 | −0.13−0.52 | 1.34 | >0.05 |
| Model 2 | Year three | −0.14 | −0.70−0.43 | 0.22 | >0.05 |
| | Year two | 0.20 | −0.14−0.54 | 1.31 | >0.05 |

BMI, body mass index; FTI, fat tissue index; LTI, lean tissue index; OH, overhydration

**Table S5 Annual variations in body composition in fall, with year one as a reference**

| **Parameters** | **Year** | **B** | **95% CI** | **Wald $\chi^2$** | ***P*** |
|---|---|---|---|---|---|
| Post-BMI | Year three | −0.38 | −0.92−0.15 | 1.95 | >0.05 |
| | Year two | 0.07 | −0.37−0.51 | 0.10 | >0.05 |
| Model 1 | Year three | −0.05 | −0.73−0.63 | 0.02 | >0.05 |
| | Year two | 0.24 | −0.26−0.74 | 0.86 | >0.05 |
| Model 2 | Year three | −0.14 | −0.80−0.53 | 0.16 | >0.05 |
| | Year two | 0.24 | −0.27−0.75 | 0.86 | >0.05 |
| Relative OH | Year three | 1.93 | 0.32−3.54 | 5.54 | 0.019 |
| | Year two | 0.81 | −0.48−2.10 | 1.50 | >0.05 |
| Model 1 | Year three | 1.81 | 0.12−3.50 | 4.40 | 0.036 |
| | Year two | 0.75 | −0.65−2.15 | 1.09 | >0.05 |
| Model 2 | Year three | 2.00 | 0.38−3.62 | 5.83 | 0.016 |
| | Year two | 0.66 | −0.68−2.00 | 0.93 | >0.05 |
| LTI | Year three | −0.13 | −0.42−0.16 | 0.79 | >0.05 |
| | Year two | −0.14 | −0.35−0.08 | 1.52 | >0.05 |
| Model 1 | Year three | −0.02 | −0.38−0.34 | 0.01 | >0.05 |
| | Year two | −0.08 | −0.32−0.16 | 0.44 | >0.05 |
| Model 2 | Year three | −0.03 | −0.36−0.31 | 0.02 | >0.05 |
| | Year two | −0.07 | −0.29−0.16 | 0.32 | >0.05 |
| FTI | Year three | −0.29 | −0.90−0.32 | 0.86 | >0.05 |
| | Year two | 0.18 | −0.35−0.71 | 0.44 | >0.05 |
| Model 1 | Year three | −0.04 | −0.80−0.72 | 0.01 | >0.05 |
| | Year two | 0.31 | −0.29−0.90 | 0.99 | >0.05 |
| Model 2 | Year three | −0.12 | −0.87−0.64 | 0.10 | >0.05 |
| | Year two | 0.29 | −0.32−0.90 | 0.89 | >0.05 |

BMI, body mass index; FTI, fat tissue index; LTI, lean tissue index; OH, overhydration

**Table S6 Annual variations in body composition in winter, with year one as a reference**

| Parameters | Year | B | 95% CI | Wald $\chi^2$ | *p* |
|---|---|---|---|---|---|
| Post-BMI | Year three | −0.46 | −0.87− −0.04 | 4.55 | 0.033 |
| | Year two | −0.29 | −0.62−0.05 | 2.86 | >0.05 |
| Model 1 | Year three | −0.18 | −0.72−0.36 | 0.42 | >0.05 |
| | Year two | −0.15 | −0.54−0.24 | 0.55 | >0.05 |
| Model 2 | Year three | −0.24 | −0.81−0.32 | 0.71 | >0.05 |
| | Year two | −0.16 | −0.55−0.24 | 0.61 | >0.05 |
| Relative OH | Year three | 1.03 | −0.56− 2.62 | 1.61 | >0.05 |
| | Year two | 0.70 | −0.68−2.08 | 0.98 | >0.05 |
| Model 1 | Year three | 1.04 | −0.53−2.62 | 1.69 | >0.05 |
| | Year two | 0.70 | −0.66−2.06 | 1.02 | >0.05 |
| Model 2 | Year three | 1.26 | −0.34−2.87 | 2.37 | >0.05 |
| | Year two | 0.75 | −0.62−2.11 | 1.16 | >0.05 |
| LTI | Year three | −0.30 | −0.62−0.02 | 3.48 | >0.05 |
| | Year two | −0.27 | −0.54−0.01 | 3.64 | >0.05 |
| Model 1 | Year three | −0.15 | −0.56−0.25 | 0.55 | >0.05 |
| | Year two | −0.21 | −0.50−0.09 | 1.87 | >0.05 |
| Model 2 | Year three | −0.22 | −0.60−0.15 | 1.41 | >0.05 |
| | Year two | −0.23 | −0.52−0.06 | 2.40 | >0.05 |
| FTI | Year three | −0.16 | −0.67−0.34 | 0.41 | >0.05 |
| | Year two | 0.00 | −0.42−0.42 | 0.00 | >0.05 |
| Model 1 | Year three | 0.01 | −0.65−0.67 | 0.00 | >0.05 |
| | Year two | 0.09 | −0.38−0.55 | 0.13 | >0.05 |
| Model 2 | Year three | −0.02 | −0.68 −0.65 | 0.00 | >0.05 |
| | Year two | 0.09 | −0.38−0.57 | 0.14 | >0.05 |

BMI, body mass index; FTI, fat tissue index; LTI, lean tissue index; OH, overhydration
